# Early salpingectomy (TUbectomy) with delayed oophorectomy to improve quality of life as alternative for risk-reducing salpingo-oophorectomy in *BRCA1/2* mutation carriers (TUBA study): a prospective non-randomised multicentre study

**DOI:** 10.1186/s12885-015-1597-y

**Published:** 2015-08-19

**Authors:** Marline G. Harmsen, Marieke Arts-de Jong, Nicoline Hoogerbrugge, Angela H. E. M. Maas, Judith B. Prins, Johan Bulten, Steven Teerenstra, Eddy M. M. Adang, Jurgen M. J. Piek, Helena C van Doorn, Marc van Beurden, Marian J. E. Mourits, Ronald P. Zweemer, Katja N. Gaarenstroom, Brigitte F. M. Slangen, M. Caroline Vos, Luc R. C. W. van Lonkhuijzen, Leon F. A. G. Massuger, Rosella P. M. G. Hermens, Joanne A. de Hullu

**Affiliations:** 1 Department of Obstetrics & Gynaecology, Radboud University Medical Center, PO Box 9101,, 6500 HB Nijmegen, The Netherlands; 2 Department of Human Genetics, Radboud University Medical Center, PO Box 9101, 6500 HB Nijmegen, The Netherlands; 3 Department of Cardiology, Radboud University Medical Center, PO Box 9101,, 6500 HB Nijmegen, The Netherlands; 4 Department of Medical Psychology, Radboud University Medical Center, PO Box 9101,, 6500 HB Nijmegen, The Netherlands; 5 Department of Pathology, Radboud University Medical Center, PO Box 9101,, 6500 HB Nijmegen, The Netherlands; 6 Department for Health Evidence, Radboud University Medical Center, PO Box 9101,, 6500 HB Nijmegen, The Netherlands; 7 Gynaecologic Oncologic Center South location Elisabeth-TweeSteden Hospital, Dr. Deelenlaan 5, 5042 AD Tilburg, The Netherlands; 8 Catharina Hospital, Michelangelolaan 2, 5623 EJ Eindhoven, The Netherlands; 9 Department of Gynaecology, Erasmus MC Cancer Clinic, ‘s-Gravendijkwal 230, 3015 CE Rotterdam, The Netherlands; 10 Center for Gynaecological Oncology Amsterdam (CGOA), Netherlands Cancer Institute/Antoni van Leeuwenhoek Hospital, Plesmanlaan 121, 1066 CX Amsterdam, The Netherlands; 11 Department of Gynaecology, University Medical Center Groningen, Hanzeplein 1, 9713 GZ Groningen, The Netherlands; 12 Department of Gynaecological Oncology, UMC Utrecht Cancer Centre, Heidelberglaan 100, 3584 CX Utrecht, The Netherlands; 13 Department of Obstetrics and Gynaecology, Leiden University Medical Centre, Albinusdreef 2, 2333 ZA Leiden, The Netherlands; 14 Department of Obstetrics and Gynaecology, Maastricht University Medical Centre, P. Debyelaan 25, 6229 HX Maastricht, The Netherlands; 15 Gynaecologic Oncologic Center South, Elisabeth-TweeSteden Hospital, Hilvarenbeekseweg 60, 5022 GC Tilburg, The Netherlands; 16 Center for Gynaecological Oncology Amsterdam (CGOA), AMC, Meibergdreef 9, 1105 AZ Amsterdam, The Netherlands; 17 Scientific Institute for Quality of Healthcare, Radboud University Medical Center, PO Box 9101, 6500 HB Nijmegen, The Netherlands

## Abstract

**Background:**

Risk-reducing salpingo-oophorectomy (RRSO) around the age of 40 is currently recommended to *BRCA1/2* mutation carriers. This procedure decreases the elevated ovarian cancer risk by 80–96 % but it initiates premature menopause as well. The latter is associated with short-term and long-term morbidity, potentially affecting quality of life (QoL). Based on recent insights into the Fallopian tube as possible site of origin of serous ovarian carcinomas, an alternative preventive strategy has been put forward: early risk-reducing salpingectomy (RRS) and delayed oophorectomy (RRO). However, efficacy and safety of this alternative strategy have to be investigated.

**Methods:**

A multicentre non-randomised trial in 11 Dutch centres for hereditary cancer will be conducted. Eligible patients are premenopausal *BRCA1/2* mutation carriers after completing childbearing without (a history of) ovarian carcinoma. Participants choose between standard RRSO at age 35–40 (*BRCA1*) or 40–45 (*BRCA2*) and the alternative strategy (RRS upon completion of childbearing and RRO at age 40–45 (*BRCA1*) or 45–50 (*BRCA2*)). Women who opt for RRS but do not want to postpone RRO beyond the currently recommended age are included as well. Primary outcome measure is menopause-related QoL. Secondary outcome measures are ovarian/breast cancer incidence, surgery-related morbidity, histopathology, cardiovascular risk factors and diseases, and cost-effectiveness. Mixed model data analysis will be performed.

**Discussion:**

The exact role of the Fallopian tube in ovarian carcinogenesis is still unclear. It is not expected that further fundamental research will elucidate this role in the near future. Therefore, this clinical trial is essential to investigate RRS with delayed RRO as alternative risk-reducing strategy in order to improve QoL.

**Trial registration:**

ClinicalTrials.gov (NCT02321228)

## Background

### BRCA germline mutations and ovarian cancer

Epithelial ovarian cancer is the most lethal malignancy of the female genital tract. With respect to treatment and prognosis, primary carcinomas of the ovaries, fallopian tubes and peritoneum are considered one disease entity often referred to as ‘ovarian carcinoma’. Women with germline mutations in one of the two *BRCA* genes are at increased risk of developing breast and ovarian cancer. Cumulative breast cancer risks are estimated 57–65 % (95 % CIs: 44–78 %) for *BRCA1* and 45–49 % (95 % CIs: 31–57 %) for *BRCA2* mutation carriers by age 70, whereas cumulative ovarian cancer risks lie around 39–40 % (95 % CIs: 18–54 %) and 11–18 % (95 % CIs: 2.4–23 %) by the age of 70 for *BRCA1* en *BRCA2*, respectively [1, 2]. Ovarian carcinoma occurs at younger age in *BRCA1* mutation carriers than in *BRCA2* mutation carriers or the general population (both mean and median 51 versus 56 versus 60 years respectively) [3]. In *BRCA1/2* germline mutation carriers, approximately 65 % of all ovarian carcinomas are of the serous subtype [4–6].

### Risk-reducing salpingo-oophorectomy (RRSO)

In contrast to breast cancer surveillance, screening for ovarian cancer has been highly ineffective [7–9]. Therefore, the only intervention to reduce ovarian cancer risk is risk-reducing salpingo-oophorectomy (RRSO), which decreases ovarian cancer incidence by about 80–96 % [4, 10–12]. However, this effect might be underestimated due to studies that included women who underwent oophorectomy alone and/or underwent surgery above the currently recommended age: 35–40 for *BRCA1* and 40–45 for *BRCA2* mutation carriers [4, 11, 13]. The residual risk of primary peritoneal cancer after RRSO is approximately 1 %; however, it was also reported to be more than 4 % [10, 14–16]. RRSO is often laparoscopically performed at an outpatients’ department. Serious surgical complications rates are low [4, 17, 18]. Main adverse effects of RRSO are related to premature surgical menopause, including short-term effects like vasomotor symptoms (i.e. hot flushes), sleep disturbances, vaginal dryness and sexual symptoms [4]. Long-term effects include osteoporosis, increased risk of cardiovascular disease, cognitive impairment and increased depressive and anxiety symptoms, although prospective studies on these long-term effects in *BRCA* mutation carriers in particular are not available [4, 19–22]. Postsurgical hormone replacement therapy (HRT) does not fully alleviate climacteric and sexual symptoms [20, 23]. The reduction of breast cancer incidence by half achieved by performing RRSO at premenopausal age [11, 12] has recently become arguable [24–26] and therefore questionable as motivation to undergo RRSO.

### Role of Fallopian tube in “ovarian” carcinogenesis

Based on recent scientific insights, the Fallopian tube is considered the most important site of origin of pelvic high grade serous carcinoma nowadays [27–31]. It is suggested that benign tubal epithelium can transform into serous tubal intraepithelial carcinoma (STIC) or invasive tubal carcinoma [32]. The (pre)malignant cells can exfoliate from the tubal epithelial lining and migrate to the ovary and abdominal cavity. This theory is based on several findings. First, no clear precursor of ovarian cancer has been found in the ovary itself. Second, earlier studies showed the presence of STIC in 36–60 % of sporadic pelvic serous carcinomas [33–35] which harboured identical mutation in the TP53 gene to the cells of concurrent pelvic serous carcinomas in 92 % [36]. Third, pelvic serous carcinoma cells resemble tubal lining epithelium more than ovarian surface epithelium [37]. Several investigators focused on prophylactically removed Fallopian tubes of germline *BRCA1/2* mutation carriers, showing the presence of STIC in about 4 % (range 0–12 %) [14, 38–47]; nearly all STICs were localised in the tubal distal fimbrial ends [34].

### Innovative preventive strategy: risk-reducing salpingectomy (RRS) with delayed oophorectomy (RRO)

The growing evidence of the role of the Fallopian tube in the origin of serous ovarian carcinoma together with the disadvantages of premature surgical menopause caused by RRSO, underlie the need for an alternative risk-reducing strategy. RRS upon completion of childbearing offers an early, potentially risk-reducing intervention; however, it is still uncertain whether and to what extent the risk of ovarian cancer will be reduced. Furthermore, around 68 % of occult carcinomas are found in tubes [48] and could now be detected at an early stage. The main advantage of delaying subsequent RRO beyond the currently recommended age will be postponement of premature menopause and its effect on noncancer-related morbidity and (menopause-related) quality of life (QoL). Several authors previously suggested this innovative strategy [48–50] and a feasibility study among both professionals and germline *BRCA1/2* mutation carriers from our group showed a broad national support to evaluate this new strategy in a prospective study [51].

### Objective

The aim of this study is to determine whether an innovative risk-reducing strategy, consisting of RRS upon completion of childbearing with delayed RRO, results in better menopause-related QoL without increase of ovarian and breast cancer risk in germline *BRCA1/2* mutation carriers compared to standard treatment, consisting of RRSO at currently recommended age.

## Methods/Design

### Study design

**Fig. 1:**
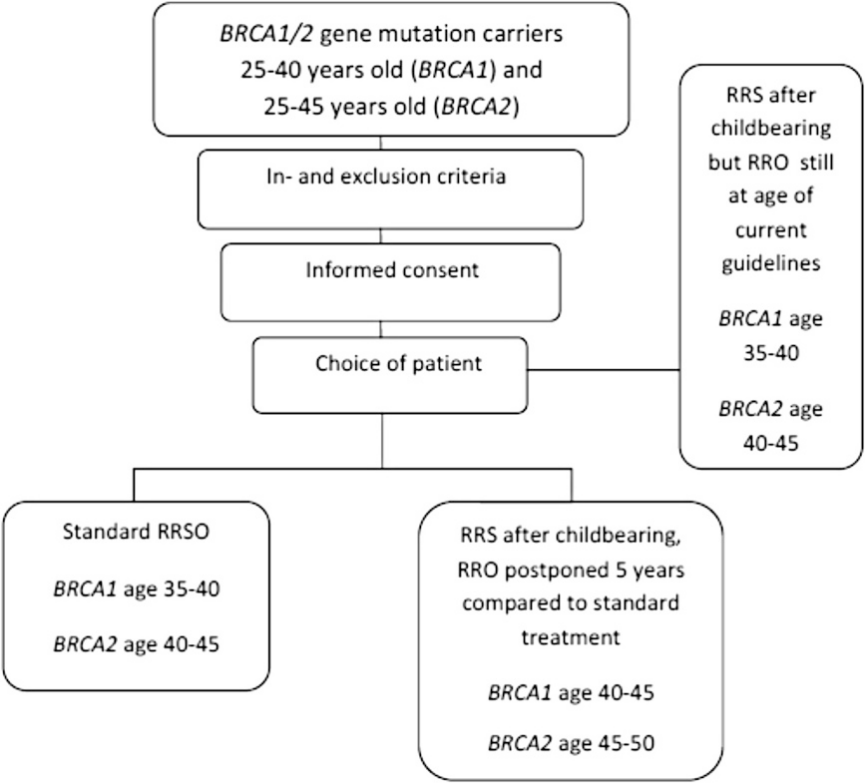
TUBA study design

We will perform a nationwide prospective non-randomised multicentre trial in 11 hospitals with a department for hereditary cancer. Eligible patients will have the opportunity to choose for standard or innovative strategy. Women who opt for RRS but do not want to postpone RRO beyond the currently recommended age or are unsure about this at enrolment are included as well; however, they will not contribute to the number of inclusions needed according to the sample size calculation. See Fig. 1 for an overview of the study design.

Although a randomised controlled trial would be the preferred study design, an earlier published feasibility study among healthcare professionals and germline *BRCA1/2* mutation carriers showed that randomisation would be an insurmountable barrier for participation in a clinical study [51]. These women want to decide themselves on their risk-reducing strategy and it is therefore unlikely that they will participate in a randomised controlled trial. Taken this into account, a prospective non-randomised design seems the most appropriate, letting women the opportunity to decide for themselves.

In addition, a control group will be formed by women who underwent RRSO between 1 and 5 years ago, in order to compare QoL between study participants and women who did not have the opportunity to choose for an alternative treatment.

### Study population

- Hospitals
  Eleven hospitals with a hereditary cancer department will participate in this study, of which seven are university tertiary hospitals.
- Patients
  Women carrying a documented germline *BRCA1/2* mutation from the department of Clinical Genetics or Hereditary Cancer of each hospital, who are between 25 and 40 (*BRCA1*) or 45 (*BRCA2*) years old without previous RRSO.
  Inclusion criteria
  Premenopausal women with a documented *BRCA1* and/or *BRCA2* gene germline mutation
  Age 25–40 years for *BRCA1* mutation carriers and 25–45 years for *BRCA2*
  Childbearing completed
  Presence of at least one Fallopian tube
  Participants may have a personal history of non-ovarian malignancy
  Informed consent
  Exclusion criteria
  Postmenopausal status (natural menopause or due to (cancer) treatment)
  Wish for second stage RRO within two years after RRS (if clear at enrolment)
  Legally incapable
  Prior bilateral salpingectomy
  A personal history of ovarian, Fallopian tube or peritoneal cancer
  Evidence of malignant disease at enrolment
  Current treatment for malignant disease
  Inability to read or speak Dutch
- Patient recruitment
  Eligible women will be sent a letter to inform them on this study. *BRCA1/2* mutation carriers will be asked to respond whether or not they would be interested to participate. If they are interested, the patient information form will be sent and an appointment will be made to explain the rationale, design and aims of the study in person. The patient will have sufficient time (minimal one week) to consider the study before deciding to participate. Written informed consent from the patient is required before participation.

Furthermore, every newly diagnosed germline *BRCA1/2* mutation carrier at the department of Clinical Genetics that fulfills the inclusion criteria will be informed on the study.

### Outcome measures

- Primary outcome measure
  Menopause-specific QoL, measured by the Greene Climacteric Scale (GCS) questionnaire in Dutch [52]. This questionnaire consists of 21 items divided into various domains: psychological (11 items, divided into anxiety and depression subscales), somatic (7 items), vasomotor symptoms (2 items) and sexual (1 item). Each symptom is rated according to its severity using a four-point Likert scale. The Greene Climacteric score is the sum of all 21 items ranging from 0 to 63. A higher total score corresponds with more menopausal symptoms.
- Secondary outcome measures
  General QoL and QoL-related items, measured by several questionnaires (see section Pre-treatment evaluation)
  Incidence of ovarian and breast cancer
  Surgical complications, e.g. infection, conversion, haemorrhage and complications at the second laparoscopic procedure (RRO) due to previous RRS.
  Histopathological findings of removed Fallopian tubes and ovaries, i.e. (pre)malignancies
  Cardiovascular risk factors and incidence of cardiovascular disease
  Cost-effectiveness of the innovative treatment compared to standard treatment

### Interventions

Standard treatment (control arm):

RRSO between age 35–40 in *BRCA1* mutation carriers and between 40–45 in *BRCA2* mutation carriers (exact ages varying across different hospitals) and when childbearing is completed.

Innovative treatment (experimental arm):

RRS when childbearing is completed with second stage RRO delayed for five years compared to the currently recommended age for RRSO, i.e. at the age of 40–45 in *BRCA1* and 45–50 in *BRCA2* mutation carriers. Regarding the definitive contraception which is a result of RRS and the age at which RRS is performed, women will be counseled in a similar manner as women consulting the gynaecologist for sterilization. RRS will be performed according to Leblanc et al*.* [53]. Whenever a (pre)malignancy is found in the RRS specimen, RRO will be performed as soon as possible, as well as additional surgery or treatment if necessary, e.g. staging procedure.

### Data collection

**Fig. 2:**
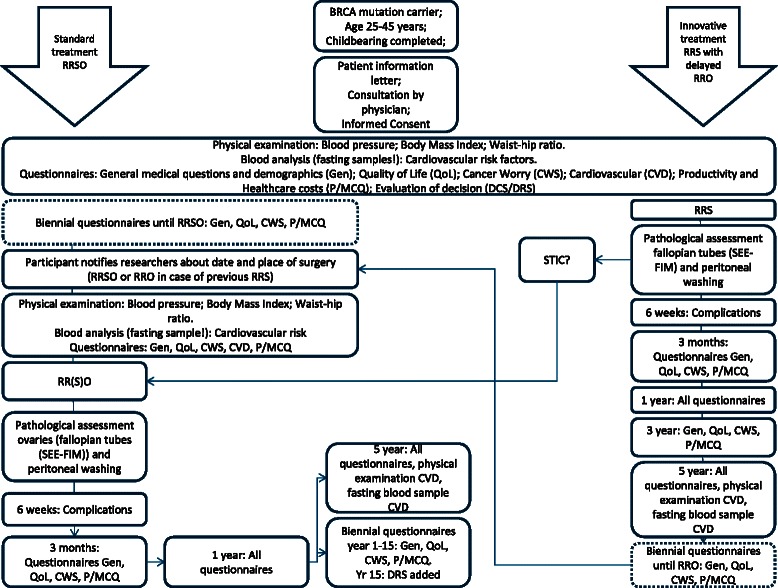
Schedule of follow-up in TUBA study
- Cost-effectiveness
  This economic evaluation will compare costs and quality adjusted life years (QALY) of the innovative treatment with standard treatment. The perspective of this economic evaluation will be a societal perspective. Both healthcare and societal costs which can be related to this study will be assessed until 15 years after last surgery. Costs are collected on a per patient level. The incremental costs of innovative care compared to standard care will be based on the difference in costs between groups. The healthcare costs, measured by the Medical Consumption Questionnaire (iMCQ) tailored to this context, will be calculated. Societal costs will be calculated from a selection of the Productivity Cost Questionnaire (iPCQ). The output or consequences of both innovative and standard care will be determined by measuring QoL before and during the study. The SF-36 and EQ-5D-5 L will be used for this analysis. The outcomes will be translated to a long-time difference in QALY. Key variables will be varied in a sensitivity analysis to evaluate their impact on the incremental costs per QALY gained ratio. Including the innovative strategy in future guideline recommendations depends on the incremental cost per QALY. As mentioned before, data will be collected until 15 years after last surgery. However, an interim cost-effectiveness analysis will be performed after eight years to provide information for proceeding implementation. Recommendation for implementation will be based on the empirical cost data completed with modelled costs over the remaining period. Accuracy of this model will be evaluated at the end of 15 years follow-up, when data collection on the actual costs during the remaining seven years will have been completed.
- Potential adverse events
  In the innovative treatment, *BRCA1/2* mutation carriers will undergo an additional laparoscopy. Known complication rates for RRSO in a comparable population vary from 0.6–5 % for major complications (conversion, bladder or bowel injury, additional surgery required) and 3.7–10 % for minor complications (infection, bleeding, haematoma) [17, 18, 42, 66]. Risks might be lower for RRS alone. As mentioned before, data on surgery-related complications will be collected six weeks after each surgery.

- Pre-treatment evaluation
  All patients will be asked to fill out web-based baseline questionnaires. Questionnaires on demographic data and medical history with a special focus on cancer and cardiovascular risk factors are included. Furthermore, QoL(−related) questionnaires include Dutch versions of the Greene Climacteric Scale (GCS) [52], SF-36 [54], EQ-5D-5 L [55], Cancer Worry Scale (CWS) [56, 57], Female Sexual Function Index (FSFI) [58, 59], Female Sexual Distress Scale (FSDS) [59, 60] and Decisional Conflict Scale (DCS) [61]. Questions based on the Institute of Medical Technology Assessment Productivity Cost Questionnaire (iPCQ) [62] and the Medical Consumption Questionnaire (iMCQ) [63] will be used to collect data on productivity loss and health consumption. Moreover, blood pressure, body mass index and waist-hip ratio will be documented. Fasting blood samples will be taken to measure cardiovascular risk factors.
- Follow-up
  Six weeks after surgery, data on surgical complications and histopathological findings are collected. The *Sectioning and Extensively Examining the FIMbriated End (SEE-FIM) of the Fallopian Tube* (SEE-FIM) protocol will be used for the latter [64]. Follow-up by web-based questionnaires as described at baseline except for the Decisional Conflict Scale is scheduled at 3 and 12 months after surgery. At 1, 5 and 15 years follow-up, the Decision Regret Scale (DRS) is added [65]. From one year after surgery, questionnaires will be sent biennially until the end of follow-up (in case of only one surgery in the standard treatment arm) or until undergoing RRO (in the innovative arm). After RRO, data will be collected at six weeks and 3 and 12 months after surgery, comparable to follow-up after the first operation, and then biennial questionnaires will be sent until the end of follow-up, 15 years after the last (or only) surgery. Additionally, blood pressure, body mass index, waist-hip ratio and cardiovascular risk factors in fasting blood samples will be collected five years after each surgery. Follow-up by questionnaires will continue biennially until 15 years after the last surgery to detect occurrence of ovarian cancer. Since the wide possible range of age at inclusion, timing of surgeries and interval between surgeries, it is hard to specify and generalize the exact amount and timing of follow-up. A flowchart visualising the follow-up schedule can be found in Fig. 2.

Furthermore, the worst-case scenario is that RRS does not reduce ovarian cancer risk at all. Then, the postponement of RRO for five years might result in a higher ovarian cancer incidence in the experimental arm. We used a model to calculate the risk for interval ovarian carcinoma when RRO is performed five years later than the current guideline age. The risk to develop ovarian carcinoma within these five years is estimated to be up to 1–2 % for *BRCA1* mutation carriers and up to 0.5–1 % for *BRCA2* mutation carriers. Cancer incidence will be monitored by questionnaires.

### Statistical analysis

- Sample size calculation
  The primary outcome measure is menopause-specific QoL. Menopausal symptoms will be assessed by the Greene Climacteric Scale (GCS). The main comparison is the difference in GCS between women getting the innovative treatment and women getting standard RRSO without postsurgical hormone replacement therapy (HRT), which is about one third of women after RRSO.
  This difference is estimated at five points on the GCS, with standard deviation 7.36, based on figures of Barentsen et al. [67]. Each hospital will provide both innovative and standard treatment, based on patient choice (no randomization). We assume an intra-cluster correlation coefficient ≤ 0.10. When we have about 10 hospitals, with 51 patients per hospital (total *n* = 510), we expect that the majority of hospitals (7 hospitals or more) will provide at least 3 patients with the innovative treatment. The remaining hospitals (3 or less) provide 51 patients with standard treatment of whom 16 will be on RRSO without HRT. This scenario gives an 80 % power (alpha = 0.05).
- Data analysis
  To test differences between two subgroups on the course of QoL since baseline, we will carry out a mixed model analysis to accommodate for hospital effects and repeated measurements. All secondary outcome measures will be analysed using mixed models in a similar manner. Cost-effectiveness, as far as it concerns the empirical data, is analyzed in a stochastic fashion using bootstrapped regression based techniques (i.e., linear mixed model) adhering to the net benefit framework.
- Safety
  An independent Data Safety Monitoring Board (DSMB) is established, existing of three independent experts who have no conflict of interest. This committee will meet once a year to perform interim analysis specifically with respect to safety. The DSMB will report to the study coordinator and may recommend changes in the conduct of the study or even premature study termination.

### Ethics

The study is conducted according to the principles of the Declaration of Helsinki (2008) and to the Medical Research Involving Human Subjects Act (Dutch: WMO). The protocol has been medical-ethically approved to be conducted in all 11 centres by the Medical-Ethical Committee of Arnhem-Nijmegen (NL 50048.091.14). The participating centres are the Radboud university medical center Nijmegen, Maastricht University Medical Centre, Erasmus MC Cancer Clinic Rotterdam, Center for Gynaecological Oncology Amsterdam (CGOA): location Netherlands Cancer Institute/Antoni van Leeuwenhoek Hospital and location Amsterdam Medical Center, University Medical Center Groningen, UMC Utrecht Cancer Centre, Leiden University Medical Centre, Gynaecologic Oncologic Center South: two locations of Elisabeth-TweeSteden Hospital Tilburg and location Catharina Hospital Eindhoven. Furthermore, the protocol is registered in Clinicaltrials.gov (NCT02321228). Written informed consent is obtained from all patients before enrolment.

## Discussion

In this study protocol, we describe a prospective non-randomised multicentre trial in premenopausal *BRCA* mutation carriers. We compare the standard strategy to reduce ovarian cancer risk, i.e. RRSO at recommended age of 35–40 in *BRCA1* and at recommended age of 40–45 in *BRCA2* mutation carriers, with an innovative risk-reducing strategy. In this innovative strategy, early RRS is performed upon completion of childbearing and subsequent RRO is delayed for five years compared to the currently recommended age for the standard strategy. The primary outcome measure is menopause-related QoL. Secondary outcome measures include safety (cancer incidence and surgical complications), histopathological findings of surgery specimens, cardiovascular risk factors and cost-effectiveness.

Currently, there are two other ongoing studies investigating different aspects of salpingectomy in germline *BRCA* mutation carriers. A research group from Texas investigates patient compliance with delayed oophorectomy after having undergone prophylactic salpingectomy (NCT01907789). They compare three regimens: ovarian cancer screening (3 years follow-up), prophylactic salpingectomy with delayed oophorectomy (4 years of follow-up including 1 year after oophorectomy) and risk-reducing salpingo-oophorectomy (1 year follow-up). QoL is measured as well. Like our study, they do not randomise. This study focuses on another endpoint, i.e. whether *BRCA* mutation carriers return for oophorectomy after earlier salpingectomy. Duration of follow-up is adjusted to this endpoint en is relatively short to assess the safety of RRS with delayed RRO as it comes to cancer incidence and non-cancer related morbidity. In our study, we focus on QoL, and several subdomains of QoL are measured as well. Nevertheless, our follow-up will not be ceased after QoL data completion, but will be prolonged to guarantee a close monitoring of cancer incidence and non-cancer related morbidity.

In a French study, *BRCA* mutation carriers who are reluctant to RRSO because of onset of premature menopause are offered a radical fimbriectomy as alternative (NCT01608074). Primary outcome is the number of pelvic serous carcinomas occurring between fimbriectomy and menopause. Secondary outcomes are perioperative morbidity, histopathologic findings of fimbriectomy specimens, incidence of breast cancer and the rate of secondary oophorectomy and associated morbidity.

In this study, fimbriectomy is only offered to women who refuse RRSO and RRS will in principle not be followed by RRO, while all women in our study eventually undergo RRO (current uptake of RRSO among *BRCA* mutation carriers is 95 % in the Netherlands). Furthermore, in this French study *BRCA* mutation carriers have to be older than 35 to be included. We include women from 25 years old, to optimize possible risk reduction by removing the Fallopian tubes as early as possible upon completion of childbearing. At last, the possible advantages of preservation of the ovaries for QoL are not evaluated in this fimbriectomy study, while this is the primary outcome in our study.

In conclusion, the current standard RRSO at age 35–40 (*BRCA1*) or 40–45 (*BRCA2*) is highly effective in reducing ovarian cancer incidence. However, consequent premature surgical menopause comes with short- and long-term noncancer-related morbidity and probably affects QoL. New insights in the origin of serous pelvic cancer put the Fallopian tube forward as target for alternative preventive surgery. The extent of the role of the Fallopian tubes in ovarian carcinogenesis remains uncertain. We expect that early salpingectomy with delayed oophorectomy is a reasonable alternative to preserve ovarian function towards the age of natural menopause without a significant increase in ovarian cancer incidence.

## Abbreviations

BRCA: Breast cancer gene
RRSO: Risk-reducing salpingo-oophorectomy
HRT: Hormone replacement therapy
(S)TIC: (Serous) tubal intraepithelial carcinoma
RRS: Risk-reducing salpingectomy
RRO: Risk-reducing oophorectomy
QoL: Quality of life
GCS: Greene Climacteric Scale
SF-36: Short-Form-36
EQ-5D: EuroQoL 5D
CWS: Cancer Worry Scale
FSFI: Female Sexual Function Index
FSDS: Female Sexual Distress Scale
DCS: Decisional Conflict Scale
DRS: Decision Regret Scale
iPCQ: Institute of Medical Technology Assessment Productivity Cost Questionnaire
iMCQ: Institute of Medical Technology Assessment Medical Consumption Questionnaire
SEE-FIM: Sectioning and Extensively Examining of the Fimbriated end
QALY: Quality adjusted life year
DSMB: Data safety monitoring board
